# Relationships Between Technology Use and Views on Aging: Results From a Randomized Controlled Trial

**DOI:** 10.1093/geroni/igaf122.1332

**Published:** 2025-12-31

**Authors:** Ines Simbrig, Felix Piazolo, Hans-Werner Wahl

**Affiliations:** UMIT TIROL – Private University of Health Sciences and Health Technology, Hall in Tirol, Austria; University of Innsbruck, Innsbruck, Tirol, Austria; Heidelberg University, Heidelberg, Baden-Wurttemberg, Germany

## Abstract

Previous research has shown that views on aging affect self-regulatory behavior and may thus also influence technology use. However, trials assessing the effect of technology use on older adults’ quality-of-life have rarely considered the role of subjective aging. Our objective was to investigate the associations between Awareness of Age-Related Change (AARC) and the use of compensatory aging-in-place technologies as part of the i-evAALution randomized controlled field study. The analytical sample contained 72 community-dwelling older adults (mean age: 76.7). 36 participants were randomly assigned to an intervention arm, using technologies like an emergency watch and a tablet with various functions, across 6-month and 13-month follow-up assessments. The 36 controls received no intervention. Primary outcomes were different quality-of-life variables, whereas AARC-Gains and -Losses were assessed as secondary outcomes. Here, we focus on the latter. Participants in the intervention group revealed a decrease in AARC-Gains during the first six months, followed by an increase up to the 13-month assessment. In contrast, participants in the control group showed an increase first, followed by a decrease. No significant difference between both conditions at the 13-month assessment and no main effect for AARC-Losses were observed. Furthermore, participants who used the emergency watch and the tablet more frequently perceived more AARC-Losses than persons who used them less frequently. Aging-in-place technologies with a strong compensatory focus may result in a dilemma such as increasing feelings of safety on the one hand, but in parallel impacting negatively the subjective experience of one’s aging.

